# Artificial intelligence to guide precision anticancer therapy with multitargeted kinase inhibitors

**DOI:** 10.1186/s12885-022-10293-0

**Published:** 2022-11-24

**Authors:** Manali Singha, Limeng Pu, Brent A. Stanfield, Ifeanyi K. Uche, Paul J. F. Rider, Konstantin G. Kousoulas, J. Ramanujam, Michal Brylinski

**Affiliations:** 1 Department of Biological Sciences, Louisiana State University, Baton Rouge, LA 70803 USA; 2 Center for Computation and Technology, Louisiana State University, Baton Rouge, LA 70803 USA; 3 Department of Pathobiological Sciences, School of Veterinary Medicine, Louisiana State University, Baton Rouge, LA 70803 USA; 4 Division of Biotechnology and Molecular Medicine, Department of Pathobiological Sciences, School of Veterinary Medicine, Louisiana State University, Baton Rouge, LA 70803 USA; 5 School of Medicine, Louisiana State University Health Sciences Center, New Orleans, LA 70112 USA; 6 Division of Electrical and Computer Engineering, Louisiana State University, Baton Rouge, LA 70803 USA

**Keywords:** Precision oncology, Cancer growth rate, Kinase inhibitors, Differential gene expression, Gene-disease association, Cancer-specific networks, Network biology, Graph neural network, Artificial intelligence, Live-cell time course assay, Gene signature

## Abstract

**Background:**

Vast amounts of rapidly accumulating biological data related to cancer and a remarkable progress in the field of artificial intelligence (AI) have paved the way for precision oncology. Our recent contribution to this area of research is CancerOmicsNet, an AI-based system to predict the therapeutic effects of multitargeted kinase inhibitors across various cancers. This approach was previously demonstrated to outperform other deep learning methods, graph kernel models, molecular docking, and drug binding pocket matching.

**Methods:**

CancerOmicsNet integrates multiple heterogeneous data by utilizing a deep graph learning model with sophisticated attention propagation mechanisms to extract highly predictive features from cancer-specific networks. The AI-based system was devised to provide more accurate and robust predictions than data-driven therapeutic discovery using gene signature reversion.

**Results:**

Selected CancerOmicsNet predictions obtained for “unseen” data are positively validated against the biomedical literature and by live-cell time course inhibition assays performed against breast, pancreatic, and prostate cancer cell lines. Encouragingly, six molecules exhibited dose-dependent antiproliferative activities, with pan-CDK inhibitor JNJ-7706621 and Src inhibitor PP1 being the most potent against the pancreatic cancer cell line Panc 04.03.

**Conclusions:**

CancerOmicsNet is a promising AI-based platform to help guide the development of new approaches in precision oncology involving a variety of tumor types and therapeutics.

**Supplementary Information:**

The online version contains supplementary material available at 10.1186/s12885-022-10293-0.

## Background

Cancer initiation and progression involve a sequence of gene-environment interaction events changing the gene expression and ultimately leading to the disruption of homeostasis [1]. The phosphorylation of various proteins is one of the key processes regulating various cellular functions, including cell cycle, apoptosis, proliferation, differentiation, growth, and others. The phosphorylation of tyrosine, serine, and threonine residues is the primary function of kinase proteins [2], 518 of which are encoded by the human genome [3]. A disruption of kinase activity can trigger the dysregulation of cellular functions and many dysregulated kinases have oncogenic effects responsible for cancer [2]. The discovery of kinase inhibitors for cancer therapy has changed the course of treatment from a conventional chemotherapy to the targeted pharmacotherapy. Although selective inhibitors are available to target certain kinases in human cancers [4], the majority of compounds bind to the highly conserved ATP binding sites of multiple targets [5–7]. Certainly, the binding promiscuity of kinase inhibitors can lead to adverse drug reactions [8–10], but also to the desired polypharmacological effects by simultaneously targeting multiple proteins involved in cancer-related processes [11–13]. Large-scale kinase inhibitor profiling experiments provide the information on the enzymatic activity inhibition across the human kinome [14, 15] greatly facilitating research on kinase-centric polypharmacological anticancer agents [16–18].

Nonetheless, the clinical efficacy of kinase-specific inhibitors is confounded by numerous factors. The success of a cancer treatment strongly depends on the underlying genetic features of the tumor, the microenvironment, the possibility of the development of drug resistance, and pharmacogenomics [8]. Numerous studies suggest that the accumulation of genetic alterations and the subsequent changes in gene expression patterns are major factors driving cancer progression [19–21]. Therefore, the identification of differentially expressed genes in various tumor types not only enhances our understanding of cancer biology [22–24], but it can also reveal new opportunities for precision oncology [25–27]. Indeed, tumor profiling with the transcriptomic analyses of gene expression networks and oncogenic pathways can increase treatment efficacy [28]. The premise of gene signature (GS)-based therapy is that an effective drug should reverse the anomalous gene expression in the disease state back to normal expression levels. Numerous resources are available to facilitate GS-based therapeutic approaches including libraries of differential gene expression for chemical perturbagens, gene knockouts, and diseases. Gene signature profiles of drug-treated and disease cells can be analyzed by various metrics of distance, similarity, anticorrelation, and those generated by machine learning models [29].

The Connectivity Map (CMap) [30] is frequently used to find connections between small molecules and diseases with the Gene Set Enrichment Analysis (GSEA) [31]. In this approach, each gene is assigned an expression value indicating to what extent it is up- or down-regulated. A disease set contains rank-ordered genes based on their differential expression against normal cells. For a given drug, a similar set of rank-ordered genes is constructed using a differential expression for drug-treated and untreated cells. Subsequently, these two lists are compared to one another to test for a negative connectivity, i.e., genes up-regulated in disease tend to be down-regulated in drug-perturbed cells and those down-regulated in disease tend to be up-regulated by the drug treatment. A strong negative connectivity indicates that treating disease cells with the drug can, in principle, restore the normal gene expression profile. On the other hand, if up- and down-regulated disease genes appear near the middle of the drug-perturbed list, one can assume that there is no connectivity between the drug and the disease, thus this treatment is unlikely to be effective.

This technique has been shown to be effective in finding new treatments for Alzheimer’s disease (AD) and glucocorticoid-resistant acute lymphoblastic leukemia (ALL) [32]. Two gene signatures, one constructed by a comparison of hippocampus from AD and the normal brain [33] and the other derived from the comparison between cerebral cortex from AD brain and age-matched controls [34], yielded a statistically significant negative connectivity with 4,5-dianilinophthalimide (DAPH) in the CMap. Indeed, a high-throughput screen of over 3000 small molecules identified DAPH as the most effective compound reversing the formation of neurotoxic fibrils associated with AD [35], followed by a synthesis of a variety of DAPH analogs as potential treatments for AD [36]. Another example is the pharmacologic modulation of glucocorticoid-resistant ALL [37]. Querying the CMap with a disease signature constructed by comparing bone-marrow leukemic cells from patients exhibiting either dexamethasone sensitivity or resistance discovered that mTOR inhibitor sirolimus can revert dexamethasone resistance. Interestingly, treating a lymphoid cell line with sirolimus significantly reduced the median inhibitory concentration (IC_50_) of dexamethasone, thus it induced the glucocorticoid sensitivity as expected [32]. Further, it was found that sirolimus sensitized tumor cells to glucocorticoid-induced apoptosis via the modulation of antiapoptotic protein MCL1 [37].

A key limitation of current drug connectivity-mapping approaches is the subjective selection of disease signatures. To address this issue, Dr. Insight implements a new statistical model utilizing the genome-wide screening of concordantly expressed genes (CEGs) [38]. Rather than extracting significantly up- and down-regulated genes from differential gene expression data, this method employs order statistics to combine drug-perturbed and disease state expression data. As a result, individual genes are assigned a concordant expression score quantifying the drug-disease connectivity and those genes having statistically significant connectivity scores are designated CEGs. The performance of Dr. Insight was evaluated against breast and prostate cancer datasets from The Cancer Genome Atlas (TCGA) [39, 40] and an additional prostate cancer dataset from the Gene Expression Omnibus (GEO) [41]. Encouragingly, Dr. Insight successfully identified fulvestrant, an FDA-approved drug against hormone receptor-positive breast cancer, and tanespimycin, alvespimycin, vorinostat, and sirolimus, which are in advanced stages of clinical trials for treating breast cancer [42]. In addition to these compounds in the ground-truth breast cancer drug list, a few novel drug treatments against breast cancer were discovered, such as 15-deoxy-delta-12,14-prostaglandin J2 inducing programmed cell death of breast cancer cells [43], and trichostatin A, a histone deacetylase inhibitor with antitumor activity against breast cancer [44].

Co-expressed GSEA can also be combined with the pathway analysis in order to infer the drug mode of action in a disease context. An example is Cogena, a pathway-guided disease and drug repositioning approach that identifies drugs acting mechanistically within the framework of coordinated changes in disease transcriptomes [45]. Cogena first performs a co-expression analysis by clustering genes showing differential expression in the disease state compared to normal cells. Subsequently, co-expressed gene clusters are subjected to a hypergeometric test against gene sets from KEGG for the pathway analysis and CMap for drug repositioning. In addition to finding new treatment opportunities, the putative drug mode of action in the disease state can be inferred from the pathway analysis and the known mode of action of a drug in the same cluster. Using the psoriatic skin transcriptome, Cogena not only successfully recovered two widely used drugs to treat psoriasis with distinct modes of action, methotrexate and ciclosporin, but it also identified several novel drugs with a high potential for repositioning to treat this disease.

The compromised drug efficacy leading to the lack of tumor response to pharmacotherapy presents notable challenges in clinical oncology. Addressing this problem requires an ability to integrate vast datasets, learn intricate relations among numerous factors, and utilize existing knowledge, surpassing the analysis of gene expression alone. Deep learning is the latest technology in the field of artificial intelligence capable of performing such complex tasks by employing sophisticated nonlinear transformations to extract patterns from high-dimensional data. Not surprisingly, deep learning has already begun to significantly impact biological and biomedical research [46]. For instance, it can help identify phenotype-related single nucleotide polymorphisms to develop accurate disease models [47], find small molecules binding to target pockets in protein structures [48–50], detect molecular targets for drugs [51], and identify opportunities for the repositioning of existing drugs to treat other conditions [52, 53].

Many recent strategies for precision oncology employ deep neural network (DNN)-based frameworks [54]. For example, a DNN trained and optimized on a pharmacogenomics database of 1001 cancer cell lines showed a high prediction accuracy against multiple clinical patient cohorts [55]. Another approach, DrugCell, is an interpretable deep learning model of human cancer cells integrating tumor genotypes with drug structure to predict response to therapy [56]. Predictions by DrugCell were shown not only to be accurate in cell lines, but also to stratify clinical outcomes. Deep learning models predicting drug response can be guided by additional data, such as signaling pathways, gene expression, and copy number variation of individual genes. Indeed, signaling pathway-constrained consDeepSignaling evaluated on the multiomics data of ∼1000 cancer cell lines was demonstrated to achieve an unparalleled performance [57]. Finally, an interpretable AI model called HiDRA (the hierarchical network for drug response prediction with attention) is capable of interpreting intrinsic characteristics of cancer cells and drugs to accurately predict cancer-drug responses [58]. This high prediction accuracy of HiDRA was attributed to paying attention to drug-target genes and cancer-related pathways when predicting a response. Despite encouraging advances in precision oncology, many existing approaches to predict the response of cancer cells to pharmacotherapy operate in the Euclidean space by utilizing various drug and cell line features. Yet, cancer initiation and development are increasingly perceived as systems-level phenomena involving intra- and inter-cellular signaling networks of the ecosystem of cancer and stromal cells [59].

To take advantage of cancer-related data having a non-Euclidean structure, we recently developed CancerOmicsNet, a graph-based deep learning system with sophisticated attention propagation mechanisms to predict the therapeutic effects of kinase inhibitors across various tumors [60, 61]. In carefully designed cross-validation benchmarks against the Library of Integrated Network-Based Cellular Signatures (LINCS) dataset [62, 63], it was shown to outperform other deep learning, graph kernel, and traditional approaches, including molecular docking and binding pocket matching. In this communication, we present the application of CancerOmicsNet to guide precision anticancer therapy with multitargeted kinase inhibitors. We first compare its performance to that of a traditional GS-based method. Next, selected predictions obtained by the application of CancerOmicsNet to “unseen” data are validated against the biomedical literature. Finally, we present the results of the experimental validation of CancerOmicsNet by live-cell time course growth rate inhibition assays for multiple drugs and tumor types not only focusing on the treatment efficacy, but also taking into account the effective drug concentration and the experimental reproducibility.

## Methods

### Benchmarking datasets for anticancer therapy

The original dataset of 3549 cell line-drug combinations involving 359 cell lines and 29 drugs was previously compiled from six LINCS-Dose-Response datasets, Broad-HMS LINCS Joint Project, LINCS MCF10A Common Project, HMS LINCS Seeding Density Project, MEP-HMS LINCS Joint Project, Genentech Cell Line Screening Initiative, and Cancer Therapeutics Response Portal [62]. These data contain drug responses in terms of GR_50_ and GR_max_ quantifying the proliferation by the value of growth rate inhibition (GR) measured in time course and endpoint assays. GR_50_ is the concentration of a drug at which GR is 0.5, whereas GR_max_ is the maximum measured GR value. Based on the sign of GR_max_, 2124 effective (negative GR_max_) and 1425 ineffective (positive GR_max_) therapies are identified. Differential gene expression profiles for the disease state were obtained from the CCLE [64]. This dataset, referred to as LINCS-3549, was used to train the deep graph learning model in CancerOmicsNet [60].

Next, we obtained drug-perturbed gene expression profiles from the CMap [30] for 107,404 combinations of 41 cell lines and 1797 small molecules, most of which have been tested at six different concentrations, 40 nm, 120 nm, 370 nm, 1.11 μm, 3.33 μm, and 10 μm. Mapping these data to LINCS-3549 resulted in 87 combinations of 11 cell lines and 24 drugs, referred to as the LINCS-87 dataset, which was employed to conduct the comparative benchmarks of CancerOmicsNet and the GS-based method. The LINCS-87 dataset comprises 40 effective (negative GR_max_) and 47 ineffective (positive GR_max_) therapies.

### “Unseen” dataset for anticancer therapy

From the Team-SKI collection of 49,348 small molecules tested against 411 protein kinases, we selected 2497 molecules absent from the LINCS growth rate inhibition dataset, thus not included in the LINCS-3549 dataset used to train CancerOmicsNet. Applying Lipinski’s rule of five [65] identified 2295 valid molecules, 288 of which are commercially available according to the ZINC library of purchasable small organic molecules [66]. Next, we selected 20 cancer cell lines from the LINCS-3549 dataset having a high balanced accuracy in the original tissue-split cross-validation benchmarks [60] and a high biomedical relevance according to a manual survey of the biomedical literature. The selected cell lines belong to 7 different tissue types, breast (HCC1428, MDAMB468, HCC70, HCC1569, HCC1937, HCC1187, HCC1395), excretory (LNCAPCLONEFGC, DU145, KMRC1, 786O), digestive (PANC0403, KYSE30, PSN1), haematopoietic and lymphoid (GRANTA519, K562), nervous (GI1, HS68), female reproductive (IGROV1), and endocrine (8505C) systems. Combining 288 commercially available kinase inhibitors with 20 cancer cell lines creates a dataset of 5760 therapies referred to as the “unseen” dataset because none of the drugs included in this dataset was used to train the machine learning model. This dataset is employed to validate CancerOmicsNet predictions.

### Gene signature-based method to predict drug response

Comparing gene signatures for drug-treated and disease cell lines is a traditional method to find potentially effective therapeutics. The GS-based method employed in this study is similar to the LINCS L1000 characteristic direction signature search engine (L1000CDS^2^) [67]. This technique utilizes the cosine distance (COS) between two types of gene expression signatures:

1 $$COS=1-\frac{\sum_{i=1}^{11113}{A}_i\ {B}_i}{\sqrt{\sum_{i=1}^{11113}{A_i}^2}\sqrt{\sum_{i=1}^{11113}{B_i}^2}}$$

where *A* is the gene signature of cancer against healthy cells and *B* is the gene signature of the same cell type before and after drug treatment. Each gene signature comprises 11,113 genes that are present in differential gene expression profiles for the disease state from the CCLE and drug-perturbed gene expression profiles from the CMap. Disease gene expression values are converted to level-5 moderated *Z*-scores [30, 67]. COS values range from 0 (signatures A and *B* are the same) to 2 (signatures A and *B* are exactly opposite). For each treatment, six COS values are calculated for all drug concentrations and the one having the longest distance is selected as a metric to predict the anticancer drug response.

### AI-based method to predict drug response

CancerOmicsNet is an AI-based system to predict a response of tumor cells to pharmacotherapy [60]. It employs a GNN model with customized graph convolution blocks and attention propagation mechanisms utilizing the cosine similarity between individual nodes. The cosine similarity quantifies a similarity of two vectors, such as node feature vectors in graphs, objects in clustering tasks, and texts in information retrieval [68], by measuring the cosine of the angle between them. The cosine measure was selected because of its low complexity and an ability to capture the semantic similarity. CancerOmicsNet was trained against the LINCS-3549 dataset and benchmarked with a tissue-level split into nine folds, digestive system, respiratory system, haematopoietic and lymphoid tissue, breast tissue, female reproductive system, skin, nervous system, excretory system, and others.

### Cell lines and culture conditions

All cell lines were maintained at 37 °C and 5% CO_2_ in a water jacketed tissue culture incubator. Pan 04.03 cells (ATCC, CRL-2555), derived from a primary tumor removed from the head-of-the-pancreas of a 70-year-old white male with pancreatic adenocarcinoma, were maintained in RPMI-1640 (ATCC, 30–2001), human recombinant insulin (20 units/mL), and 15% fetal bovine serum. HCC70 cells (ATCC, CRL-2315), isolated from a primary ductal carcinoma from a 49-year-old black female, were maintained in RPMI-1640 (ATCC, 30–2001) and supplemented with 10% fetal bovine serum. DU 145 cells (ATCC, HTB-81), derived from a 69-year-old white male with prostate cancer, were maintained in Eagle’s minimum essential medium (EMEM) (ATCC, 30–2003) and supplemented with 10% fetal bovine serum.

### Lentivirus transduction

Incucyte nuclight red lentivirus reagent (Sartorius, Catalogue No. 4476) was purchased and used to transduce Pan 04.03, DU 145, and HCC70 cell lines at a multiplicity of infection (MOI) or 1. Briefly, 3 × 10^5^ cells were seeded into one well of a 6 well plate (Corning, Catalogue No. 353046). After an overnight incubation, 200 μL of lentivirus particles consisting of ~ 3 × 10^5^ transducing units were applied to the cells and returned to the incubator overnight. The following day, the media was replaced and cells were allowed to expand for 3 days. Next, cells were selected for transduction with the addition of 1 μg/mL puromycin. Nuclear fluorescence was visualized on an inverted fluorescent microscope maintained and under continual selection for further analysis.

### Drugs

JNJ-7706621 (MedChemExpress, HY-10329), PP1 (MedChemExpress, HY-13804), AZD6482 (MedChemExpress, HY-10344), XMD8–93 (MedChemExpress, HY-14443), GW2580 (MedChemExpress, HY-10917), and PI-103 (MedChemExpress, HY-10115) were purchased from suppliers and resuspended to a stock concentration of 10 mm in DMSO.

### Live-cell time course inhibition assay

Cells were seeded at a density of 5000 cells/well in 384 well plates (Corning, Catalogue No. 3764) in duplicate wells containing 20 μL media and incubated overnight. The following day, 20 μL of a 2× dilution series was applied to the cells to produce the final concentrations of 1 nm, 3.162 nm, 10 nm, 31.62 nm, 100 nm, 316.2 nm, 1 μm, 3.162 μm, and 10 μm. Cells were then imaged for 72 hours with the IncuCyte S3 system at 400 ms acquisition time in the red channel and the 10× objective. Adherent cell-by-cell analysis was conducted to quantify the number of red nuclei in each well over the 72-hour observation period. The entire experiment was repeated after a week; we refer to the first series of measurements as experiment A and the second series as experiment B.

### Cell count

For any sample, including drug-treated and control groups, at any time *t* during the 72-hour observation period, a normalized cell count, *N*_*norm*_, is calculated as:

2 $${N}_{norm}(t)=\frac{N(t)}{N\left({t}_0\right)}\times 100$$

where *N*(*t*) is the number of red nuclei and *N*(*t*_0_) is the initial number of red nuclei recorded at the outset of measurements. This way, the normalized initial number of cells across all experiments is always 100. In addition to the normalized cell count, a relative cell count for the drug-treated group with respect to the control, *N*_*rel*_, is calculated as:

3 $${N}_{rel}\left(d,c,t\right)=\frac{N_{norm}\left(d,c,t\right)}{N_{norm}\left( ctrl,t\right)}$$

where *N*_*norm*_(*d*, *c*, *t*) is the normalized number of red nuclei for the group treated with a drug *d* at concentration *c*, and *N*_*norm*_(*ctrl*, *t*) is the normalized number of red nuclei for the same cell line in DMSO measured at the same time *t*.

### Growth rate calculation

Following the original paper describing the growth rate formalism [62], a *GR* value for a drug *d* at concentration *c* and time *t* is calculated as:

4 $$GR\left(d,c,t\right)={2}^{\frac{{\mathit{\log}}_2\left(N\left(d,c,t+\Delta t\right)/N\left(d,c,t-\Delta t\right)\right)}{{\mathit{\log}}_2\left(N\left( ctrl,t+\Delta t\right)/N\left( ctrl,t-\Delta t\right)\right)}}-1$$

where *N*(*d*, *c*, *t* ± Δ*t*) is the cell count for the group treated with drug *d* at concentration *c* and time *t* ± Δ*t* while *N*(*ctrl*, *t* ± Δ*t*) is the cell count for the control group at time *t* ± Δ*t*. Δ*t* is chosen as 6 hours according to the original work [62]. For each experiment involving a cell line and a drug at a certain concentration, a series of GR values are calculated at different time points and the minimum numerical GR value is selected as the GR_max_ (max stands for the maximum efficiency).

## Results

### Overview of CancerOmicsNet

**Fig. 1:**
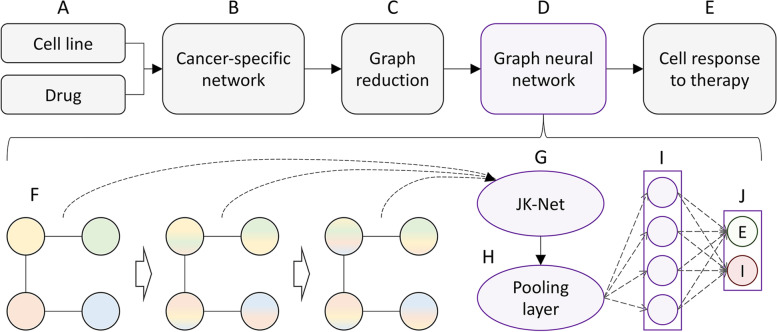
Flowchart of CancerOmicsNet. **A** The input consists of a cancer cell line and a drug. **B** The graph representation of the input data integrating a PPI network, differential gene expression, kinase inhibitor profiling, and gene-disease associations. **C** A graph reduction procedure guided by the topological information and the biological knowledge. **D** The graph neural network utilizing attention propagation mechanisms. **E** The predicted response of the cell line to the drug treatment. **F** A series of customized graph convolution blocks to generate node embeddings with information propagation steps represented by hollow arrows. The information initially carried by individual nodes is shown in different colors. **G** The JK-Net combining node embeddings produced by individual blocks. **H** A global pooling layer integrating node embeddings into the graph embedding. **I** A set of fully connected layers. **J** An output layer predicting the cell response to the drug treatment (E – effective, I – ineffective)

CancerOmicsNet utilizes an integrated graph representation of multiple heterogeneous data, including biological networks, pharmacogenomics, kinase inhibitor profiling, and gene-disease associations [61]. The flowchart of CancerOmicsNet is presented in Fig. 1. Input data, a cancer cell line and a kinase inhibitor (Fig. 1A), are used to obtain a differential gene expression profile from the Cancer Cell Line Encyclopedia (CCLE) [64], disease-gene associations from DISEASES [69] and DisGeNET [70], and the kinase inhibitor profile from Team-SKI [71]. These data are integrated and mapped onto the human protein-protein interaction (PPI) network from STRING [72] to build a cancer-specific network for a given combination of a cell line and a drug (Fig. 1B). Subsequently, the full-size network is subjected to a reduction procedure driven by the biological knowledge to construct a compact, information-rich graph increasing the feature entropy and preserving the valuable graph-feature information (Fig. 1C) [61]. The reduced network is then utilized by a graph neural network (GNN) with sophisticated attention propagation mechanisms (Fig. 1D) to predict the therapeutic effect of the input drug on the cell line of interest (Fig. 1E).

The GNN model contains a series of customized graph convolution blocks (Fig. 1F) to generate node embeddings. Each block utilizes a cosine similarity-based attention mechanism to better direct the information flow between nodes. The information carried by individual nodes in the graph is represented by different colors in Fig. 1F. After each propagation step (hollow arrows) nodes receive the information from their neighbors. The information from different convolution blocks is aggregated by a jumping knowledge network (JK-Net) designed to combine node embeddings produced by individual blocks into a single embedding for each node (Fig. 1G) [73]. The JK-Net can be viewed as an attention mechanism for different convolution layers yielding a significant performance boost. Next, the Set2Set pooling layer [74] is employed to integrate the embeddings of all nodes into the graph embedding accounting for the lack of node order in the graph (Fig. 1H). Finally, the graph embedding is passed through a series of fully connected layers (Fig. 1I) to predict the outcome of the drug treatment (Fig. 1J), either effective (E) or ineffective (I).

### Comparative benchmarks of GS- and AI-based methods

We first compare the performance of CancerOmicsNet to that of a traditional approach employing the gene signature analysis. As a GS-based method, we implemented an algorithm similar to the L1000CDS^2^ search engine prioritizing small molecule signatures for their predicted ability to either reverse or mimic gene expression in a disease state [67]. This method utilizes the cosine distance (COS) between the gene signatures of disease cells and drug-treated cells. COS values larger than 1 indicate that a drug can reverse the disease state, so the treatment is predicted to be effective. In contrast, COS values less than 1 indicate that a drug treatment mimics the disease state, therefore it is unlikely to be effective.

Table 1 shows the performance of CancerOmicsNet and the GS-based method against the LINCS-87 growth rate inhibition dataset. CancerOmicsNet clearly outperforms the gene signature analysis, especially looking at the accuracy (ACC) and the area under the receiver operating characteristic curve (AUC-ROC). Although the GS-based method yields high precision (PPV), which is the fraction of effective treatments among the retrieved instances, the recall (TPR) quantifying the fraction of effective treatments that were retrieved is low. These results indicate that even though those treatments predicted by the analysis of gene signatures to be effective are usually correct, the majority of effective treatments remain undetected. Contrastingly, CancerOmicsNet yields not only a much higher prediction accuracy for the same dataset, but the results are overall more robust compared to the GS-based approach.

**Table 1:** Performance of the gene signature (GS)-based method and CancerOmicsNet in detecting effective anticancer treatments**.** Recall (TPR), precision (PPV), accuracy (ACC), F_1_ score, and the area under the receiver operating characteristic curve (AUC-ROC) are calculated for a dataset of 87 treatments

Method	TPR	PPV	ACC	F_**1**_ score	AUC-ROC
GS-based	0.447	0.950	0.437	0.608	0.475
CancerOmicsNet	0.714	0.712	0.714	0.712	0.761

In order to better illustrate the concept of the GS-based prediction of drug efficacy, we discuss two representative examples selected from the benchmarking dataset. The first example is an ATP-competitive protein tyrosine kinase inhibitor dasatinib [75] impeding the growth of the breast adenocarcinoma cell line MCF7 with a half-maximal growth inhibitory concentration (GI_50_) of 1.6 μm [76]. Dasatinib is effective against MCF7 with a GR_max_ of − 0.07, which is indicative of a cytotoxic response. The COS distance is 1.08, therefore the GS-based method correctly predicted the sensitivity of MCF7 to dasatinib. The second example is a selective JAK1 and JAK2 inhibitor ruxolitinib [77] and the skin melanoma cell line A375 with a GR_max_ value of − 0.25. Ruxolitinib is in phase 2 of a clinical trial against squamous cell skin cancer [78]. The GS-based method incorrectly predicted the treatment of A375 with ruxolitinib to be ineffective based on the COS distance of 0.96 between drug-perturbed and disease gene signatures.

**Fig. 2:**
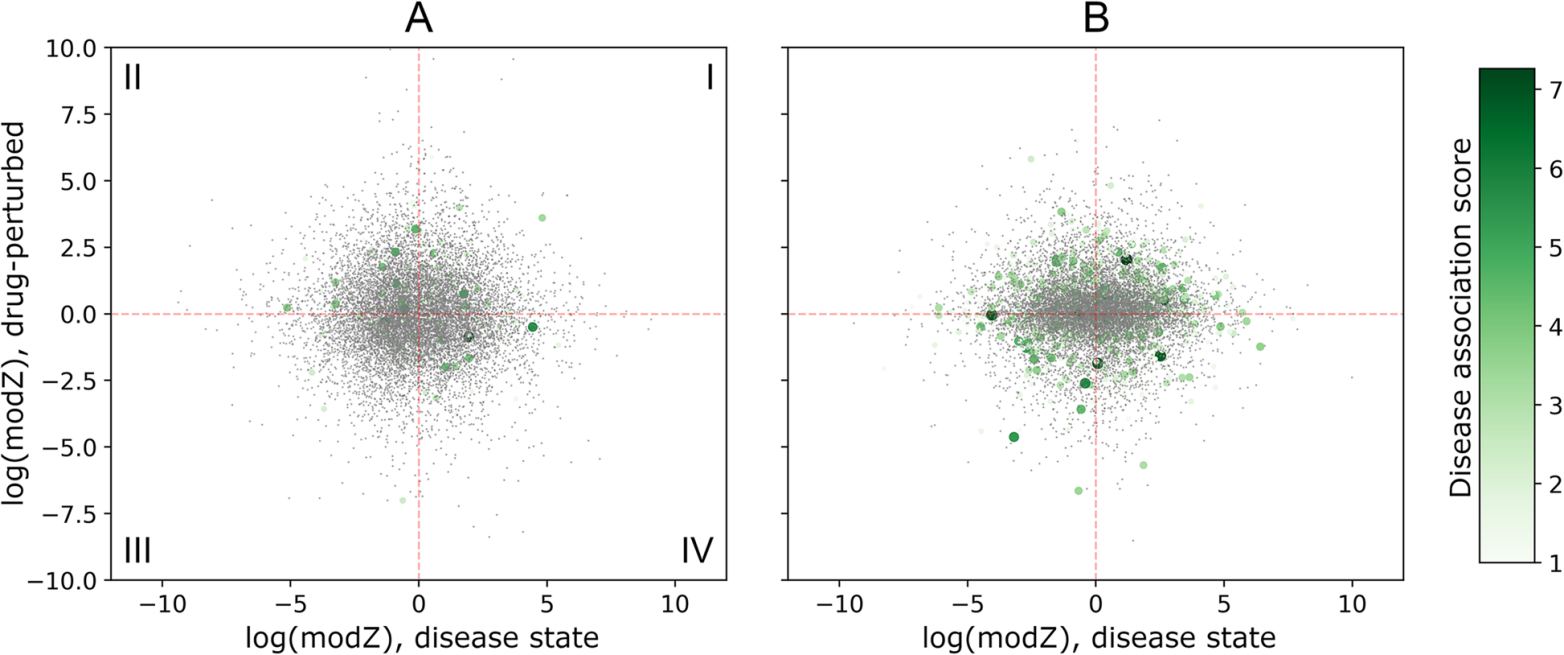
Gene signature-based approach to predict the outcome of anticancer drug treatment. **A** The cell line MCF7 and dasatinib and **B** the cell line A375 and ruxolitinib. Each dot represents a gene whose differential expression in the disease state is on the abscissa and drug-perturbed differential expression is on the ordinate. Differential expression values are computed as the moderated *Z*-score (modZ) weighted averages of replicated level-5 gene signatures. Those genes having disease associations with breast adenocarcinoma in **A** and skin melanoma in **B** are shown as larger circles colored according to the scale on the right. Each plot is divided into quadrants, which are labeled I-IV in **A**

Figure 2 shows the scatter plots of moderated *Z*-score (modZ) values computed for gene expression in cancer cell lines and those obtained for the drug treatment. Since the GS-based approach predicts effective treatments when drugs can potentially reverse the gene expression state of cancer cells, one would expect to find most genes in quadrants II and IV in Fig. 2. This is not the case because the fractions of genes in quadrants I, II, III, and IV are, respectively, 0.22, 0.25, 0.28, and 0.25 for dasatinib and MCF7 (Fig. 2A), and 0.27, 0.26, 0.22, and 0.23 for ruxolitinib and A375 (Fig. 2B). We also mapped disease association scores to individual genes according to the color scale shown in Fig. 2. Interestingly, the sum of scores for genes in quadrants II and IV (752.1) is higher than for genes in quadrants I and III (731.5) for the treatment of MCF7 with dasatinib that was correctly predicted by the GS-based analysis to be effective. For the treatment of A375 with ruxolitinib, incorrectly predicted to be ineffective, the sum of scores in quadrants II and IV (1568.0) is lower than in quadrants I and III (1664.5).

**Fig. 3:**
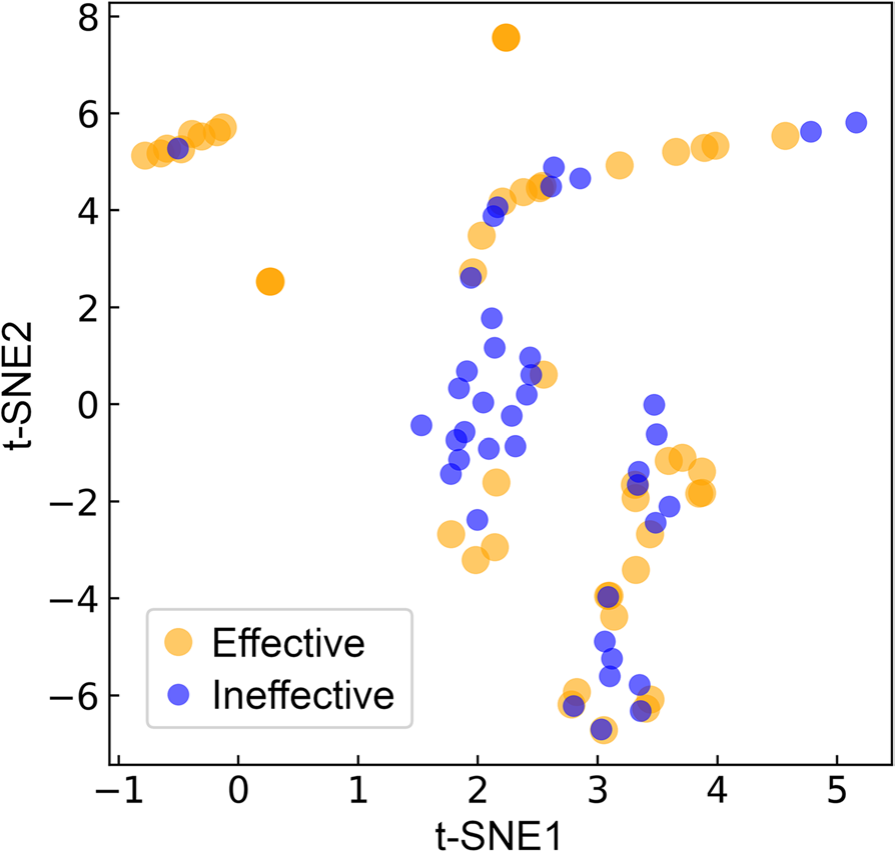
Separation of the output layer graph embeddings of CancerOmicsNet in a low-dimensional space. The T-distributed Stochastic Neighbor Embedding (t-SNE) technique is applied to 40 effective (blue) and 47 ineffective (gold) treatments in the LINCS-87 growth rate inhibition dataset

In contrast to the GS-based approach, AI-based CancerOmicsNet correctly predicted both treatments, MCF7 with dasatinib and A375 with ruxolitinib, to be effective with probabilities of 0.99 and 0.65, respectively. AI models are specifically designed to learn complex patters from the input data in order to make accurate predictions. To better understand the performance of machine learning in detecting effective treatments, high-dimensional graph embeddings from the output layer of CancerOmicsNet can be visualized in a two-dimensional space with t-distributed stochastic neighbor embedding (t-SNE), a nonlinear dimensionality reduction technique [79]. Figure 3 shows the visualization of 40 effective (blue) and 47 ineffective (gold) treatments from the LINCS-87 growth rate inhibition dataset. The t-SNE algorithm models the data such that similar instances are close to one another, while dissimilar instances are far away from each other. Indeed, groups of neighboring points in Fig. 3 contain predominantly either effective or ineffective treatments, which is consistent with the high accuracy of CancerOmicsNet in predicting the outcome of anticancer treatment.

### Literature-based validation of CancerOmicsNet

**Fig. 4:**
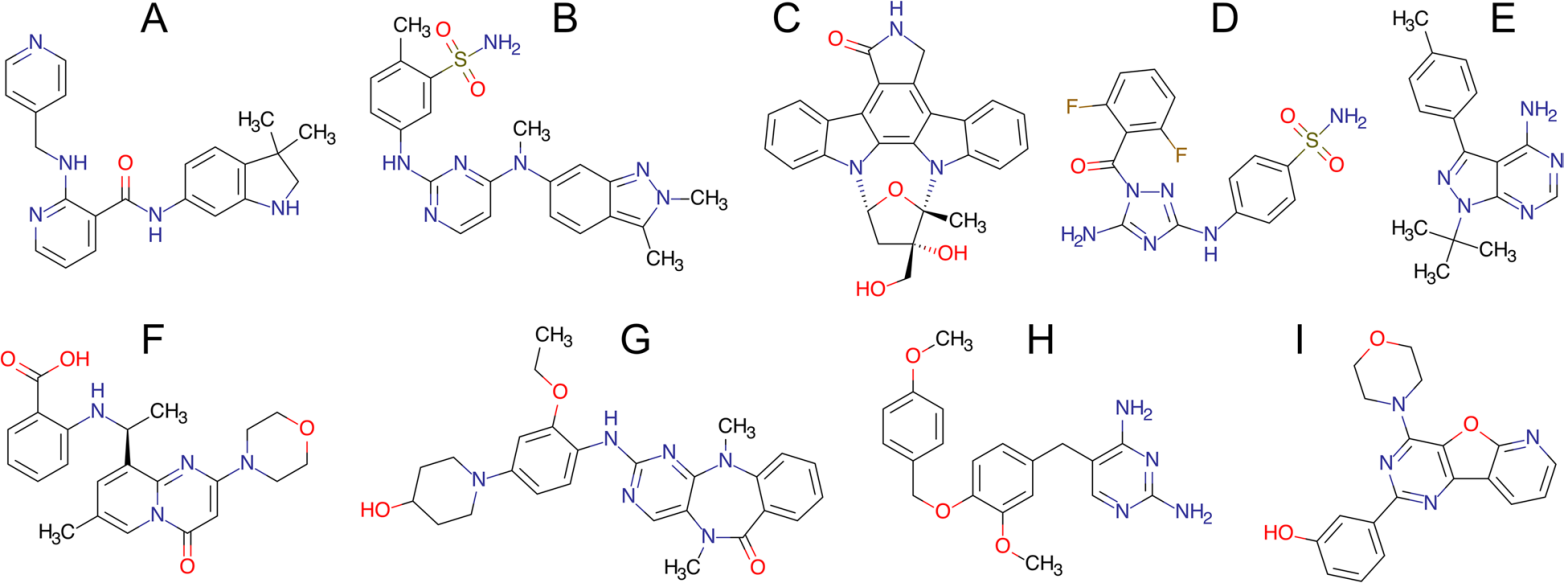
Drugs selected for literature-based and experimental validation of CancerOmicsNet predictions. **A** AMG 706, **B** GW786034, **C** CEP-701, **D** JNJ-7706621, **E** PP1, **F** AZD6482, **G**, XMD8–92, **H** GW2580, and **I** PI-103

We discuss the performance of CancerOmicsNet in several cases of “unseen” data, viz. treatments absent from the LINCS-3549 growth rate inhibition dataset that was originally used to train the machine learning model. Each novel prediction is supported by the evidence found in the biomedical literature. The structures of drugs selected for the literature-based validation are presented in Fig. 4A-C. The first molecule is motesanib (AMG 706, Fig. 4A), an anthranilamide inhibitor of vascular endothelial growth factor receptors (VEGFR) with IC_50_ values of 2 ± 0.7 nm (VEGFR1), 3 ± 0.5 nm (VEGFR2), and 6 ± 4 nm (VEGFR3) [80]. Although VEGFR kinases are its primary targets, motesanib also inhibits the activity of platelet derived growth factor receptor beta (PDGFRβ) at an IC_50_ of 84 ± 33 nm, mast/stem cell growth factor receptor Kit (c-KIT) at an IC_50_ of 8 ± 2 nm, and tyrosine-protein kinase receptor Ret (c-RET) at an IC_50_ of 59 ± 4 nm [81]. This drug has been tested alone and in combination with chemotherapy in human non-small-cell lung cancer xenograft models created by injecting NCI-H358, NCI-H1299, NCI-H1650, A549, and Calu-6 cancer cell lines subcutaneously into mice. Tested against A549 at three different concentrations, 7.5, 25, and 75 mg/kg b.i.d, motesanib inhibited the tumor growth by 45, 84, and 107%, respectively. CancerOmicsNet estimated a high probability of 0.82 for the growth inhibition of A549 cell line by motesanib. Further, the tumor growth of Calu-6 xenograft was inhibited by 66% at the highest tested dose of motesanib [82]. Encouragingly, the probability that motesanib inhibits the growth of Calu-3 cell line reported by CancerOmicsNet is as high as 0.97. Note that according to the Cellosaurus [83], Calu-3 (originated from a 25-year-old male) and Calu-6 (originated from a 61-year-old female) are closely related lung adenocarcinoma cell lines.

Motesanib also has antitumor activity against breast cancer [80]. Its primary targets, VEGFR proteins, are angiogenic factors that modulate processes playing important roles in the development and progression of breast cancer [84]. Motesanib was tested against MCF-7, MDA-MB-231, and Cal-51 xenografts of breast cancer. It inhibited MCF-7 tumor growth by 44% at a concentration of 25 mg/kg and by 65% at a concentration of 75 mg/kg. Further, motesanib inhibited MDA-MB-231 tumor growth by 64% at the highest concentration. Cal-51 tumor growth was also reduced by 38, 74 and 81% when the drug was administered at 7.5 mg/kg, 25 mg/kg and 75 mg/kg, respectively [84]. CancerOmicsNet estimated that the probabilities of inhibiting the growth of MCF-7, MDA-MB-231, and Cal-51 breast cancer cell lines are 0.88, 0.95, and 0.93, respectively.

Pazopanib (GW786034, Fig. 4B) inhibits intracellular tyrosine kinases, PDGFRα with an IC_50_ of 73 nm, PDGFRβ with an IC_50_ of 215 nm, VEGFR1 with an IC_50_ of 7 nm, VEGFR2 with an IC_50_ of 15 nm, and VEGFR3 with an IC_50_ of 2 nm [85]. It exhibits antiangiogenic properties and it is used to treat renal cell carcinoma (RCC) [86]. Pazopanib was tested in 8 human RCC cell lines, 769-P, 786-O, HRC-24, HRC-31, HRC-45, HRC-78, RCC-26B, and SK-45, showing a varying degree of antiproliferative activities [87]. For instance, it reduces the proliferation of 786-O cell lines by 50% at > 100 μm. According to CancerOmicsNet, the probability of inhibition of the 786-O cell line growth by pazopanib is 0.76. Pazopanib was also tested alone and in combination with topotecan against anaplastic thyroid cancer (cell line 8305C) [88], one of the most aggressive, but rare forms of thyroid cancer. 72 hours after the treatment with pazopanib, the proliferation of 8305C cell line was inhibited at an IC_50_ of 25 ± 3.2 μm. According to the Cellosaurus, 8305C (originated from a 67-year-old female) and 8505C (originated from a 78-year-old female) cell lines are closely related anaplastic thyroid cancers and CancerOmicsNet estimated that pazopanib inhibits the growth of 8505C with a high probability of 0.93.

Lestaurtinib (CEP-701, Fig. 4C) is a multitargeted kinase inhibitor structurally related to staurosporine [89]. It inhibits FMS-like tyrosine kinase 3 (FLT3) with an IC_50_ of 2 to 3 nm [90], Janus kinase 2 (JAK2) with an IC_50_ of 1 nm [91], and tyrosine receptor kinases (Trk) with an IC_50_ of 100 nm [92]. Human pancreatic ductal adenocarcinoma (PDAC) shows an aberrant expression of neurotrophin and its associated Trk receptors [93]. After the drug was administered at 10 mg/kg b.i.d into a mouse model created by subcutaneously injecting a PDAC cell line Panc1, the growth of the xenograft showed a significant decrease with a *p*-value of < 0.01 [93]. CancerOmicsNet predicted with a high probability of 0.98 that lestaurtinib inhibits the growth of Panc 04.03, which is a closely related PDAC cell line.

### Experimental validation of CancerOmicsNet

**Fig. 5:**
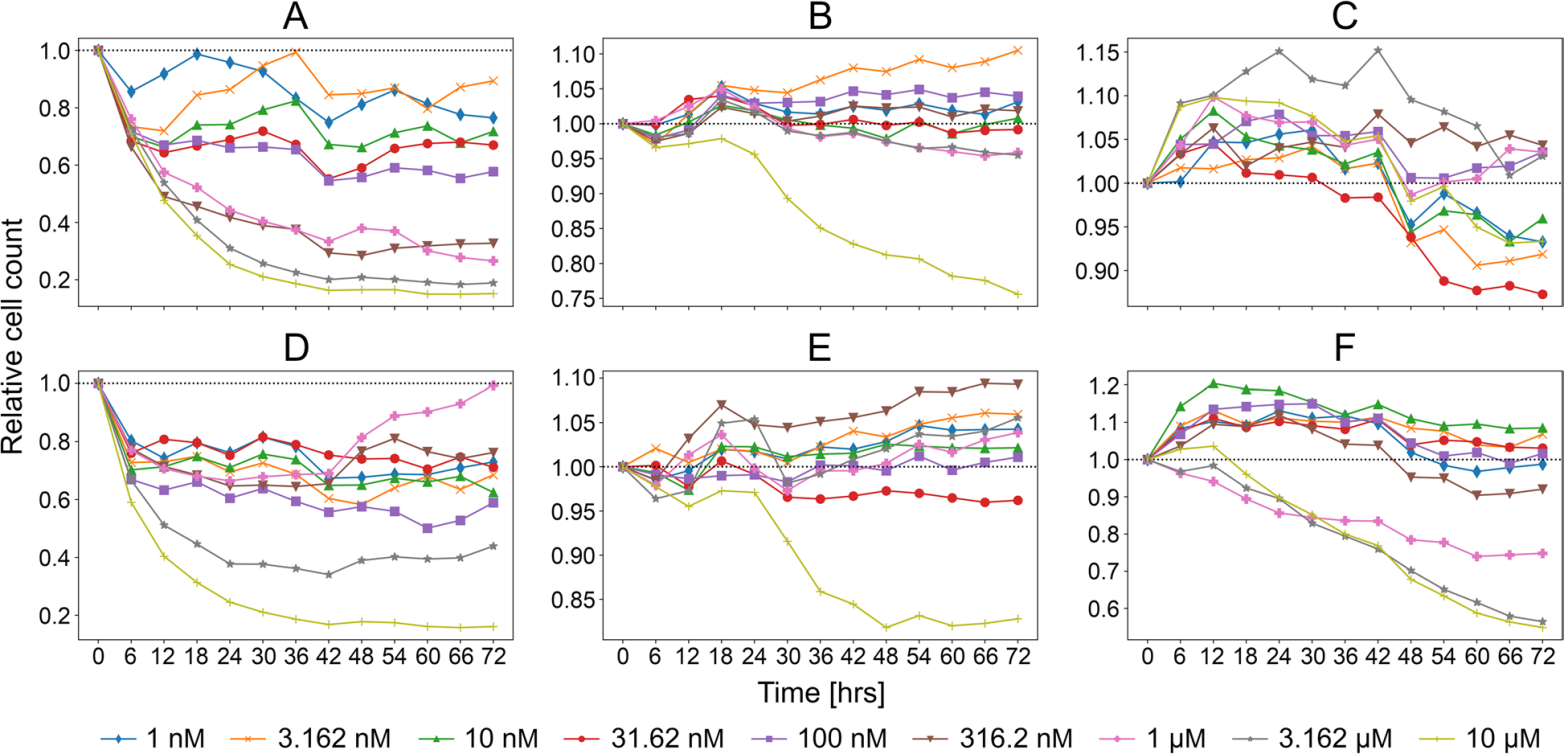
Time courses of relative cell counts after a drug treatment in different concentrations. Panc 04.03 treated with (**A**) JNJ-7706621 and (**D**) PP1, DU 145 treated with (**B**) AZD6482 and (**E**) XMD8–92, and HCC70 treated with (**C**) GW2580 and (**F**) PI-103

Selected predictions by CancerOmicsNet for the “unseen” data were subjected to experimental validation by live-cell time course inhibition assay. Eight drugs, whose structures are presented in Fig. 4D-I, have been tested at nine different concentrations, ranging from 1 nm up to 10 μm. The measured relative cell counts are shown in Fig. 5, whereas the corresponding GR_max_ values are reported in Table 2. The first two drugs are JNJ-7706621 (Fig. 4D) and PP1 (Fig. 4E). JNJ-7706621 is a pan-CDK inhibitor, which also potently inhibits Aurora kinases A and B [94]. It exhibits an antiproliferative activity against several cell lines, A-375 (melanoma) with an IC_50_ of 447 nm, HCT116 (colorectal carcinoma) with an IC_50_ of 254 nm, and HeLa (Human papillomavirus-related endocervical adenocarcinoma) with an IC_50_ of 284 nm [95]. Another study of JNJ-7706621 reports IC_50_ values of 286 ± 72 nm (HeLa), 189 ± 42 nm (HCT116), 410 ± 75 nm (SK-OV-3, ovarian cancer), 112 ± 12 nm (PC-3, prostate cancer), 416 ± 54 nm (A-375), 514 ± 63 nm (MDA-MB-231, triple-negative breast cancer), 263 ± 113 nm (DU 145, prostate cancer), and 413 ± 4 nm (MES-SA, uterine sarcoma) [94]. PP1 is a potent and selective Src inhibitor for LCK and Fyn kinase proteins [96] that has been tested against the acute megakaryoblastic leukemia cell line M-07e at 100 nm, 500 nm, 1 μm, 2.5 μm, and 5 μm concentrations [97]. PP1 inhibited the stem cell factor (SCF) induced proliferation with an IC_50_ of 0.5–1 μm, whereas 2.5 μm completely prevented the SCF-induced proliferation of M-07e cells.

**Table 2:** Experimental validation of drug treatment predictions by CancerOmicsNet. GR_max_ values obtained for three cell lines treated with six drugs at various concentrations. For each treatment, two values collected from experiment A / experiment B are reported

Drug concentration	Panc 04.03		DU 145		HCC70	
	***JNJ-7706621***	***PP1***	***AZD6482***	***XMD8–92***	***GW2580***	***PI-103***
1 nm	−1.000 / -0.596	−1.000 / -0.771	− 0.344 / -0.147	− 0.791 / -0.998	− 0.086 / -0.361	0.176 / -0.077
3.162 nm	−1.000 / -0.807	− 1.000 / -0.830	− 0.908 / -0.968	− 0.892 / -0.992	0.129 / -0.040	0.042 / 0.036
10 nm	− 1.000 / -0.922	− 1.000 / -0.697	− 0.788 / -0.429	− 0.929 / -0.998	0.245 / 0.022	− 0.472 / -0.265
31.62 nm	−1.000 / -0.708	− 1.000 / -0.257	− 0.609 / -0.995	− 0.962 / -0.996	− 0.131 / -0.048	− 0.274 / 0.176
100 nm	− 1.000 / -0.818	− 0.999 / -0.908	− 0.977 / -0.991	− 0.840 / -0.999	− 0.421 / 0.335	0.158 / -0.076
316.2 nm	−0.111 / -0.968	− 1.000 / -0.759	− 0.340 / -0.977	− 0.557 / -0.976	−0.116 / 0.556	− 0.353 / -0.437
1 μm	− 0.570 / -0.982	−1.000 / -0.838	−0.581 / -0.982	− 0.892 / -0.996	0.139 / 0.249	0.238 / -0.330
3.162 μm	−0.026 / -0.665	−1.000 / -0.738	−0.211 / -0.998	− 0.799 / -0.993	−0.206 / -0.345	− 0.943 / -0.884
10 μm	− 0.188 / -0.723	−1.000 / -0.739	−0.987 / -0.995	− 0.714 / -0.852	−0.808 / -0.319	− 0.985 / -0.797

**Fig. 6:**
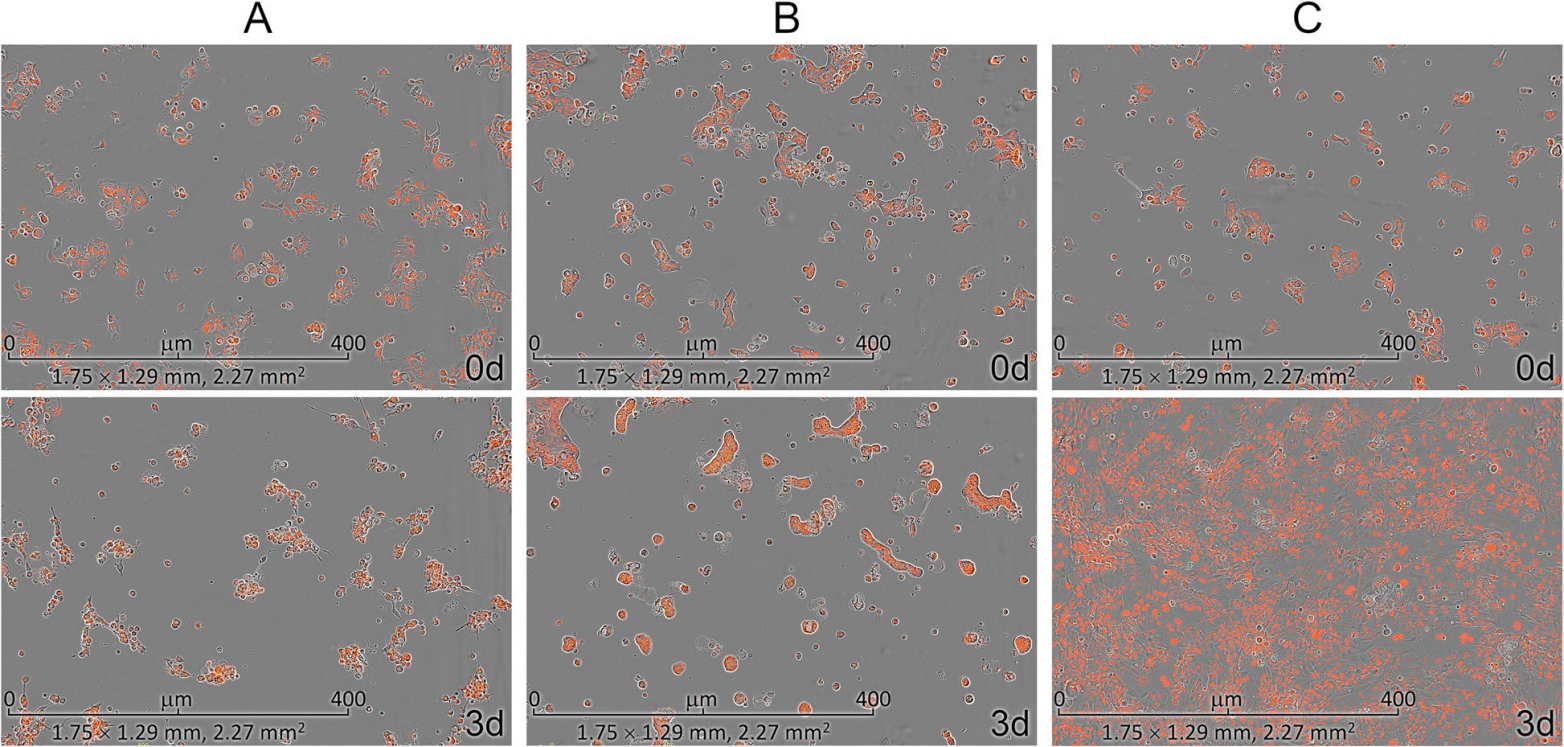
Microscopy images of Panc 04.03 cells. The treatment with (**A**) JNJ-7706621 at 10 μm and (**B**) PP1 at 10 μm is compared to (**C**) the vehicle treatment. The first row shows images recorded before the treatment and the second row shows images taken 3 days after the treatment

Interestingly, CancerOmicsNet predicted that both compounds are effective against the pancreatic adenocarcinoma epithelial cell line Panc 04.03 with a high confidence of 0.93 for JNJ-7706621 and 0.91 for PP1. Figure 6 shows fluorescent microscopy images recorded in experiment A for the treatment of Panc 04.03 cells with JNJ-7706621 (Fig. 6A) and PP1 (Fig. 6B), both at 10 μm concentration, compared to the control group consisting of vehicle (DMSO)-treated cells (Fig. 6C). Experiments started with a normalized initial number of 100 cells (the first row of Fig. 6). Three days after cells have been treated with drugs, the normalized cell counts were 110 for JNJ-7706621 and 117 for PP1 (the second row of Fig. 6A and B, respectively). The second row of Fig. 6C shows that the control group significantly proliferated in 3 days to the normalized cell count of as high as 725. Further, Fig. 5A (JNJ-7706621) and 5D (PP1) show that the relative cell counts calculated against the control group systematically decrease after the treatment in a concentration-dependent manner. For instance, 3 days after Panc 04.03 cells were treated with JNJ-7706621 at 1 nm, 10 nm, 100 nm, 1 μm, and 10 μm, the relative cell counts are 0.76, 0.72, 0.58, 0.27, and 0.15, respectively (Fig. 5A). In addition to the time course of relative cell counts, Table 2 reports GR_max_ values calculated for JNJ-7706621 and PP1 against Panc 04.03 in two experiments, A and B, carried out at a one-week interval. Encouragingly, negative GR_max_ values show that these drugs are effective at all concentrations, clearly inhibiting the proliferation of Panc 04.03 cells with respect to the vehicle-treated control group.

The next two compounds are AZD6482 (Fig. 4F) and XMD8–92 (Fig. 4G). The former drug is a selective PI3Kβ inhibitor with an IC_50_ of 0.69 nm [98]. Tested in various PTEN-deficient cancer cell lines including breast (HCC70, MDA-MB-468, and BT-549) and prostate (PC3) cancers, AZD6482 was demonstrated to efficiently inhibit the tumor growth by strongly impairing the PI3K signaling [99]. XMD8–92 is a potent and selective dual inhibitor of big map kinase (BMK1, ERK5) and bromodomain-containing proteins (BRDs, BET) with a *K*_d_ of 80 nm for ERK5 and 170 nm for BRD4 [100]. This compound was profiled against a diverse panel of tumor types, exhibiting an antiproliferative activity with EC_50_ values in the single-digit micromolar range against prostate (PC-3 and BPH-1), brain (SK-N-AS) and non-small cell lung (NCI-H1299 and NCI-H522) cancer cell lines [101]. CancerOmicsNet predicted that AZD6482 and XMD8–92 should also be effective against the human prostate cancer cell line DU 145 with confidence indices of 0.73 and 0.79, respectively. The time courses plotted in Fig. 5B for AZD6482 and 5E for XMD8–92 show that although treated cells initially continue to proliferate, the relative cell counts drop below the 1.0 threshold for higher drug concentrations. For example, 3 days after the cells were treated with AZD6482 and XMD8–92 at 10 μm, the relative cell counts are 0.76 and 0.83, respectively. Further, GR_max_ values calculated for AZD6482 and XMD8–92 reported in Table 2 show that both drugs are systematically effective against the DU 145 cell line.

The last two drugs are GW2580 (Fig. 4H), a selective CSF1R inhibitor of c-FMS with an IC_50_ of 30 nm [102], and PI-103 (Fig. 4I), a multi-targeted PI3K inhibitor of p110α/β/δ/γ with an IC_50_ of 2 nm/3 nm/3 nm/15 nm [103]. Although GW2580 strongly inhibited the growth of freshly isolated human monocytes with an IC_50_ of 330 ± 50 nm, the growth of human foreskin fibroblasts, endothelial cells, and five tumor cell lines (breast MDA-MB-231 and BT-474, lung A549, head/neck HN-5, and gastric NCI-N87) was highly resistant to GW2580 [102]. Tested in three different glioblastoma cell lines containing PTEN mutations, PI-103 was demonstrated to block PI3K signaling and inhibit the proliferation of U-118 MG at 60 nm, U-87 MG at 600 nm, and U-138 MG at 1.0 μm [104]. CancerOmicsNet predicted that both compounds are effective against the human triple-negative mammary carcinoma cell line HCC70 with a confidence of 0.76 for GW2580 and 0.74 for PI-103. When these drugs are administered at higher concentrations, the relative cell counts drop below the 1.0 threshold, for instance, the relative cell count is 0.93 and 0.55 3 days after the cells were treated with GW2580 and PI-103, respectively, both at 10 μm. Further, GR_max_ values reported in Table 2 show that these compounds, depending on the concentration, can inhibit the proliferation of the HCC70 cell line.

### Experimental reproducibility

**Fig. 7:**
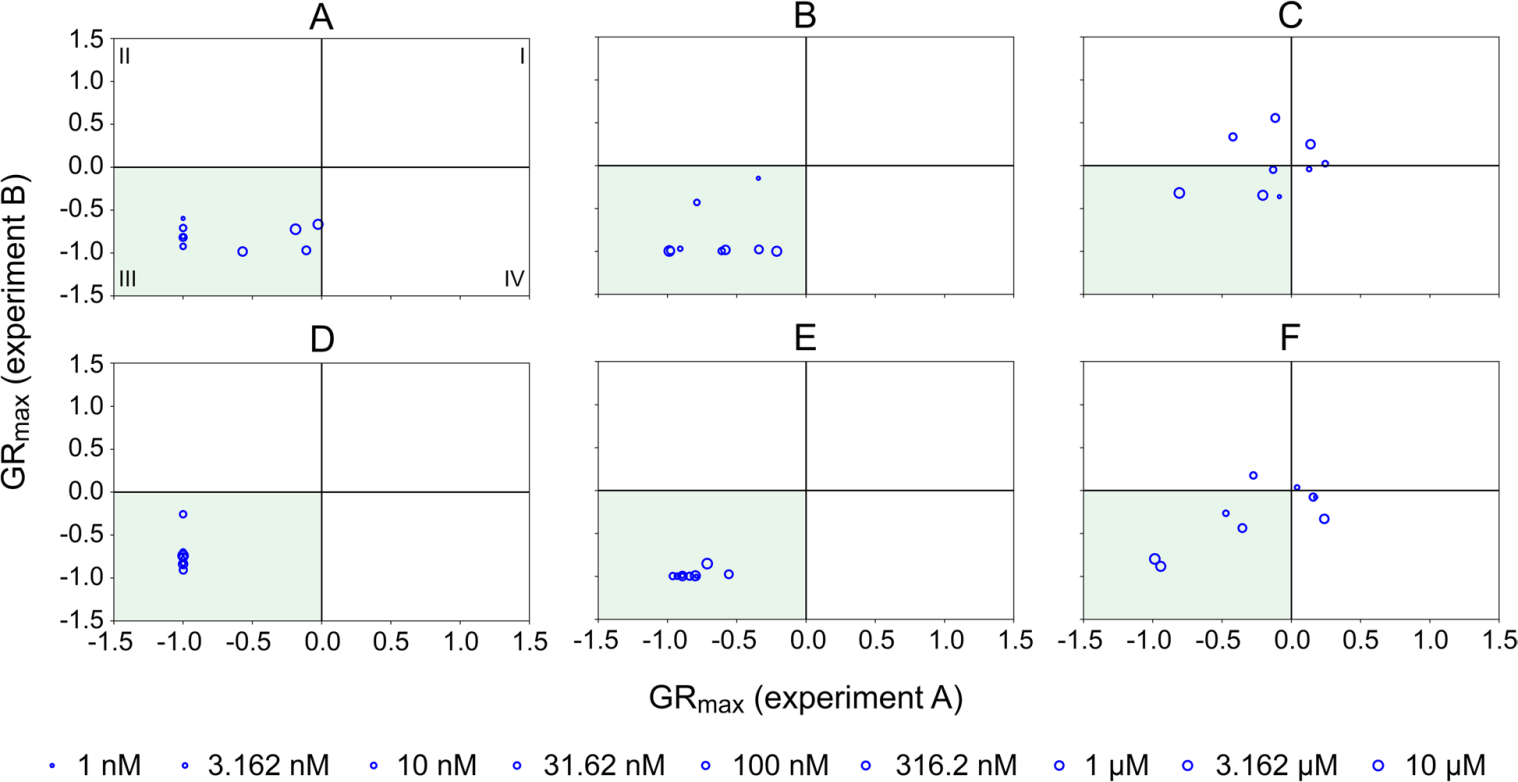
Correlation between GR_max_ values obtained from experiments A and B. Panc 04.03 treated with (**A**) JNJ-7706621 and (**D**) PP1, DU 145 treated with (**B**) AZD6482 and (**E**) XMD8–92, and HCC70 treated with (**C**) GW2580 and (**F**) PI-103. The size of each blue circle corresponds to the drug concentration. Each plot is divided into quadrants, which are labeled I-IV in **A**

The experimental validation of CancerOmicsNet predictions was conducted in two series of growth rate inhibition assays, referred to as experiments A and B, carried out at a one-week interval. Figure 7 shows correlation plots for GR_max_ values collected from these two experiments. In order to help evaluate the consistency between different experiments, each plot is divided into quadrants, labeled I-IV in Fig. 7A, according to the sign of GR_max_ indices calculated from the data collected in each series of experiments. Encouragingly, most data points are in quadrant III (colored green in Fig. 7) encompassing drug concentrations with negative GR_max_ values in both experiments, meaning that these compounds systematically inhibited the proliferation of cancer cells. A few points in quadrant I in Fig. 7C and F represent the concentrations of GW2580 and PI-103 with positive GR_max_ values observed in both experiments against the HCC70 cell line. Lastly, data points in quadrants II and IV in Fig. 7C and F correspond to those concentrations of GW2580 and PI-103 inhibiting the proliferation of HCC70 cells only in one out of two experiments. Nonetheless, these points not only represent low drug concentrations and are close to borderlines with quadrants I and III, but there is also a noticeable correlation between GR_max_ values collected against the HCC70 cell line in experiments A and B. Overall, both validation experiments yielded consistent results positively validating CancerOmicsNet predictions.

### Effective drug concentration

**Fig. 8:**
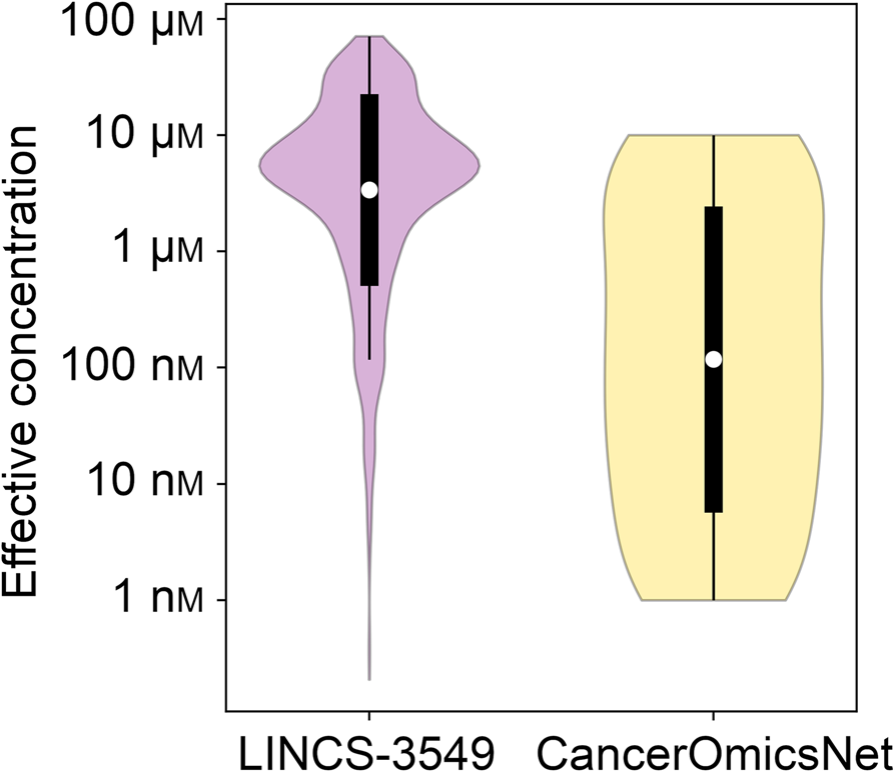
Analysis of the effective drug concentration. Drug concentrations resulting in negative GR_max_ values are considered effective. The distribution of concentrations across the LINCS-3549 growth rate inhibition dataset is compared to those measured experimentally for CancerOmicsNet predictions

Finally, we conducted a statistical analysis of effective drug concentrations measured in validation experiments in comparison to those obtained from the LINCS-3549 growth rate inhibition dataset. Here, a drug concentration is considered effective when the corresponding GR_max_ value is negative. Figure 8 shows that 54.2% combinations of cancer cell lines and anticancer compounds in the LINCS-3549 dataset have effective concentrations with a mean ± standard deviation of 9.4 ± 12.1 μm. Encouragingly, 71.3% cases of CancerOmicsNet predictions tested experimentally show negative GR_max_ values with a mean ± standard deviation effective concentration of 1.8 ± 3.3 μm. Therefore, most drugs predicted by CancerOmicsNet as effective against target cancer cell lines not only exhibit the desired anticancer activity, but also tend to be effective at lower concentrations compared to those publicly available in a large database of anticancer agents tested against various tumors.

## Discussion

CancerOmicsNet is a recently developed system utilizing the AI technology to guide precision oncology. Benchmarked against a large dataset of anticancer therapies comprising multitargeted kinase inhibitors and a wide variety of tumor types, it was previously demonstrated to outperform many other approaches, including deep learning methods, graph kernel models, molecular docking, and drug binding pocket matching [60]. In this communication, the performance of CancerOmicsNet is also compared to data-driven therapeutic discovery utilizing a popular concept of “signature reversion” that aims at molecules able to reverse disease-specific gene expression patterns [105]. Despite many examples of the successful application of GS-based methods reported to date, salient issues with this methodology remain unsolved [106].

For instance, the expression of landmark genes carefully selected by the LINCS consortium may not necessarily reflect the mechanism of action of therapeutic candidates [107]. We also noticed this problem when analyzing disease and drug-perturbed gene expression profiles for two representative therapies in our dataset, dasatinib-breast adenocarcinoma and ruxolitinib-skin melanoma. Even though both therapies are effective, no clear indication of the ability of these drugs to reverse disease expression patterns was observed. Further, we found that the approach utilizing the gene expression analysis yields a high precision at a low recall. While the identified molecules tend to be effective, most treatments in the dataset are undetected. Much of the success in GS-based therapeutic discovery seems to be strongly contingent on the manual curation of a set of signature genes for a specific medical condition [108]. Indeed, including gene-disease association scores for breast adenocarcinoma more plausibly described the efficacy of dasatinib in the context of signature reversion.

In general, methods employing the AI technology predict cellular responses to pharmacotherapy with a better accuracy. In our benchmarking calculations, using CancerOmicsNet yields more robust discrimination between effective and ineffective anticancer treatments compared to the GS-based approach. This improved performance of machine learning can be attributed to the utilization of multiple biomedical data, including PPI networks, gene expression patterns, gene-disease associations, and kinase inhibitor profiling. In addition, employing deep learning enables CancerOmicsNet to automatically extract meaningful features in order to effectively learn complex patterns present in these data. Interestingly, since deep learning models are not explicitly instructed which patterns to look for in the data, they may pick up on associations among multiple variables that are not easily perceptible. We expect that integrating more biological data will further increase the accuracy of anticancer treatment prediction.

The promise of CancerOmicsNet in precision oncology is comprehensively investigated by evaluating its ability to generalize to “unseen” data comprising 288 kinase inhibitors with no growth rate inhibition values in LINCS. None of these molecules has been used to train the deep learning model, hence the term “unseen” data. The effect of these drugs on the growth of several cell lines predicted by CancerOmicsNet was first validated against the biomedical literature. Encouragingly, those inhibitors assigned high probabilities to reduce the proliferation of certain cell lines have been reported to exhibit the predicted anticancer activities in independent studies. Finally, six compounds were validated experimentally in live-cell time course assays against breast, pancreatic, and prostate cancer cell lines. The tested molecules exhibited dose-dependent antiproliferative activities with negative GR_max_ values in most concentrations. In particular, pan-CDK inhibitor JNJ-7706621 and Src inhibitor PP1 were the most potent against the pancreatic cancer cell line Panc 04.03. Validation experiments repeated after 1 week yielded consistent results. It is also noteworthy that, on average, anticancer drugs predicted by CancerOmicsNet were found effective in lower concentrations than active molecules in the LINCS database. We note that antiproliferative properties can be predicted for any compound that has been profiled against a panel of human kinases with respect to its inhibitory potency and selectivity.

Similar to other methods to predict therapeutic effects, CancerOmicsNet has several limitations. One obvious complication is the selection of drug efficacy measure, such as GR_50_, GR_max_, and IC_50_. These measures often depend on the experimental setup with respect to drug concentrations and the duration of measurements [107]. For instance, some molecules may work better in higher concentrations and after a longer time than the specific range of concentration and duration selected for the experiment. Further, GR values are calculated based on cell count differences between the future time stamp and the previous time stamp, with an underlying assumption that cancer cells in the control group proliferate continuously. This can be problematic for some cell lines that may be difficult to grow under conditions selected for the experiment. Other issue arises from the cancer heterogeneity, which can result in different drug efficacies measured for the same tumor types [109].

Future directions in the development of CancerOmicsNet include the integration of other large-scale cancer data, such as the single nucleotide polymorphism and the mutation information, which would facilitate a more personalized selection of anticancer therapies based on the tumor genetic makeup. Moreover, utilizing data related to the molecular mechanisms of metastatic cells governing their mobility and plasticity would allow for the prediction of other therapeutic effects, such as the inhibition of cancer cell viability, migration, and invasion ability. We also plan to conduct a pharmacophore analysis of molecules exhibiting antiproliferative activities and expand the repertoire of therapeutics outside the range of kinase inhibitors. Including other drug classes will make it possible to extend CancerOmicsNet to predict effective combinations of molecules having different mechanisms of action. Synergistic effects are generally highly beneficial allowing the use of lower doses of the combination constituents often leading to significantly reduced adverse reactions [110]. Overall, the results of comprehensive benchmarking calculations, experimentally validated predictions, and numerous opportunities for further improvements and extensions make CancerOmicsNet a promising AI-based platform to guide precision oncology with a broad range of applications involving a variety of cancer types and therapeutics.

## Supplementary Information

**Additional file 1: Supplementary Video 1.** Live-cell time course recording of Panc 04.03 cell line treated with JNJ-7706621 at 10 μm.

**Additional file 2: Supplementary Video 2.** Live-cell time course recording of Panc 04.03 cell line treated with PP1 at 10 μm.

**Additional file 3: Supplementary Video 3.** Live-cell time course recording of Panc 04.03 cell line treated with DMSO as the control.

**Additional file 4: Supplementary Video 4.** Live-cell time course recording of DU 145 cell line treated with AZD6482 at 10 μm.

**Additional file 5: Supplementary Video 5.** Live-cell time course recording of DU 145 cell line treated with XMD8–92 at 10 μm.

**Additional file 6: Supplementary Video 6.** Live-cell time course recording of DU 145 cell line treated with DMSO as the control.

**Additional file 7: Supplementary Video 7.** Live-cell time course recording of HCC70 cell line treated with GW2580 at 10 μm.

**Additional file 8: Supplementary Video 8.** Live-cell time course recording of HCC70 cell line treated with PI-103 at 10 μm.

**Additional file 9: Supplementary Video 9.** Live-cell time course recording of HCC70 cell line treated with DMSO as the control.

## Data Availability

CancerOmicsNet is open sourced and freely available to the academic community at https://github.com/pulimeng/CancerOmicsNet. Comparative benchmarking results for the LINCS-87 dataset and CancerOmicsNet predictions for “unseen” data are available from the Open Science Framework at https://osf.io/kv3wa/.

## Abbreviations

ACC: Accuracy
AD: Alzheimer’s disease
AI: Artificial intelligence
ALL: Acute lymphoblastic leukemia
AUC-ROC: Area under the receiver operating characteristic curve
CCLE: Cancer Cell Line Encyclopedia
CEGs: Concordantly expressed genes
COS: Cosine distance
CMap: The Connectivity Map
DMSO: Dimethyl sulfoxide
EMEM: Eagle’s minimum essential medium
GEO: Gene Expression Omnibus
GI_50_: Half-maximal growth inhibitory concentration
GNN: Graph neural network
GR: Growth rate
GS: Gene signature
GSEA: Gene Set Enrichment Analysis
IC_50_: Half-maximal inhibitory concentration
JK-Net: Jumping knowledge network
L1000CDS^2^: L1000 characteristic direction signature search
LINCS: Library of Integrated Network-Based Cellular Signatures
modZ: Moderated *Z*-score
MOI: Multiplicity of infection
PDAC: Pancreatic ductal adenocarcinoma
PDGFR: Platelet derived growth factor receptor
PPI: Protein-protein interaction
PPV: Positive predictive value or precision
RCC: Renal cell carcinoma
SCF: Stem cell factor
t-SNE: t-Distributed stochastic neighbor embedding
TCGA: The Cancer Genome Atlas
TPR: True positive rate or recall
Trk: Tyrosine receptor kinase
VEGFR: Vascular endothelial growth factor receptor
